# Cost per Responder Analysis of Secukinumab versus Adalimumab in the Treatment of Psoriatic Disease

**DOI:** 10.3390/vaccines10050646

**Published:** 2022-04-20

**Authors:** Paolo Gisondi, Davide Geat, Martina Maurelli, Luca Degli Esposti, Francesco Bellinato, Giampiero Girolomoni

**Affiliations:** 1 Section of Dermatology and Venereology, Department of Medicine, University of Verona, 37126 Verona, Italy; davide.geat@univr.it (D.G.); martina.maurelli@studenti.univr.it (M.M.); francesco.bellinato@univr.it (F.B.); giampiero.girolomoni@univr.it (G.G.); 2 CliCon S.r.l. Health, Economics & Outcomes Research, 40121 Bologna, Italy; luca.degliesposti@clicon.it

**Keywords:** cost per responder, secukinumab, adalimumab

## Abstract

Background: The EXCEED study evaluated the efficacy and safety of secukinumab versus adalimumab in psoriatic arthritis, but it did not include a pharmacoeconomic analysis. The objective of this study was to compare the cost per responder of secukinumab versus adalimumab in patients with psoriatic disease. Methods: The cost per responder was calculated by multiplying the cost of treatment by the number needed to treat for each therapy. The 52-week primary endpoint was the American College of Rheumatology response rate (ACR) 20; secondary endpoints were ACR 50, Psoriasis Area and Severity Index (PASI) 90, and minimal disease activity (MDA). Results: The cost per responder for ACR 20 was €19,846 versus €19,766 for secukinumab and adalimumab, respectively, whereas the costs per responder for ACR 50 and PASI 90 were €27,820 versus €27,384 and €22,102 versus €32,375 for secukinumab and adalimumab, respectively. The cost per MDA responder was €34,072 and €38,906 for secukinumab versus adalimumab. Conclusions: The costs per responder associated with the psoriatic arthritis end points were similar for adalimumab and secukinumab; conversely, the costs for psoriasis and composite end points were lower for secukinumab.

## 1. Introduction

Psoriasis is a common chronic immune-mediated disease that affects 1–4% of the population worldwide, and about 14 million people in Europe [1]. About 20–30% of psoriatic patients have a moderate-to-severe disease [2] and are candidates for systemic treatments such as phototherapy, conventional systemic agents (acitretin, ciclosporin, methotrexate, fumarates), and targeted therapies (biologics and small molecules) [3]. Of note, the treatment of moderate-to-severe psoriasis with biologic agents poses a significant economic burden to health care systems [4]. Approximately 20–25% of patients affected by psoriasis also develop psoriatic arthritis (PsA), a chronic disease that affects peripheral and axial joints and entheses, typically after the onset of the skin manifestations [5]. Psoriasis can also be associated with several comorbidities other than PsA, including cardiovascular diseases, metabolic syndrome, inflammatory bowel diseases, and psychiatric diseases such as depression, anxiety, and suicidal ideation [6,7,8,9,10]. Both psoriasis and PsA have a relevant negative impact on patients’ quality of life. In a National Psoriasis Foundation survey, psoriasis and PsA affected overall emotional wellbeing in 88% of patients and interfered with enjoyment of life in 82% [11]. Furthermore, patients with severe psoriasis have 1.8 times greater odds of being unemployed compared to patients with mild psoriasis [11]. Among biological disease-modifying antirheumatic drugs (bDMARDs), secukinumab and adalimumab, which belong to the classes of IL-17A and TNF-α inhibitors, respectively, are two agents approved for the treatment of both plaque psoriasis and PsA [12]. The efficacy and the safety of secukinumab and adalimumab as first-line biological monotherapy in patients with psoriatic disease was evaluated in the EXCEED study, a head-to-head randomized controlled trial that included patients naïve to bDMARDs and intolerant or with an inadequate response to conventional systemic disease-modifying antirheumatic drugs (csDMARDs) [13,14]. The EXCEED study did not include a pharmacoeconomic analysis. The objective of this study was to compare the cost per responder of secukinumab versus adalimumab in patients with psoriatic disease from the perspective of the Italian National Health System.

## 2. Materials and Methods

A cost per responder analysis of secukinumab versus adalimumab was developed based on the efficacy data from the EXCEED study (Table 1) [13,14]. The EXCEED study was a double-blind, parallel-group, randomized, active-controlled, phase IIIb trial that enrolled 853 patients with active PsA and concomitant plaque psoriasis. In particular, 426 patients received secukinumab and 427 adalimumab for 52 weeks. The efficacy was measured using the American College of Rheumatology (ACR 20, 50, and 70) criteria, Psoriasis Area and Severity Index (PASI 75, 90, and 100), and minimal disease activity (MDA) response rate at week 52 (Table 1). MDA is a combined joint and skin outcome measure. MDA is defined as that state of disease activity deemed a useful target of treatment by both the patient and physician, given current treatment possibilities and limitations. A patient achieves MDA when five of the following seven criteria are met: tender joint count ≤ 1; swollen joint count ≤ 1; Psoriasis Area and Severity Index ≤ 1 or body surface area ≤ 3%; patient pain visual analog score (VAS) ≤ 15; patient global disease activity VAS ≤ 20; Health Assessment Questionnaire (HAQ) Disability Index ≤ 0.5; tender entheseal points ≤ 1 [15]. The 52-week primary endpoint was the ACR 20, and the secondary endpoints were ACR 50 and PASI 90. Secukinumab 300 mg was administrated by subcutaneous (S.C.) injections at weeks 0, 1, 2, 3, and 4 followed by 300 mg every 4 weeks. Adalimumab 40 mg was injected by a S.C. initial dose of 80 mg, followed by 40 mg every other week, beginning one week after initial dose (Table 2). 

### Cost per Responder Model

The cost per responder model was based on the perspective of the Italian National Health System. Regarding the costs of biologic drugs, ex-factory wholesale purchase prices were used, including the mandatory discounts according to the national legislation (5% discount, plus a further 5% reduction on the discount result) (Table 3) [16,17]. The 2022 costs were reported in Euros. Only drug acquisition costs were considered, while other costs including treatment administration and monitoring were not included. The cost per responder was calculated by multiplying the cost of treatment by the number needed to treat (NNT) for each of the therapies. The NNT is the inverse of the absolute risk reduction (ARR). The ARR is the absolute difference in the rates of events (such as PASI 75/90/100, ACR 20/50/70, MDA) between secukinumab relative to adalimumab (i.e., secukinumab event rate (SER) minus the adalimumab event rate (AER), ARR = SER − AER). Scenario analyses with different discount rates (in addition to the mandatory discounts) were performed (Supplementary Tables S2–S8).

Each package of secukinumab (Cosentyx^®^, Novartis Farma S.p.A, Largo U. Boccioni 1—21040 Origgio (VA), Italy) includes two syringes; each package of adalimumab (Humira^®^, Abbvie Italia S.r.l., S.R. 148 Pontina Km 52 snc 04011 Campoverde di Aprilia (LT), Italy) includes two syringes. 

## 3. Results

**Figure 1:**
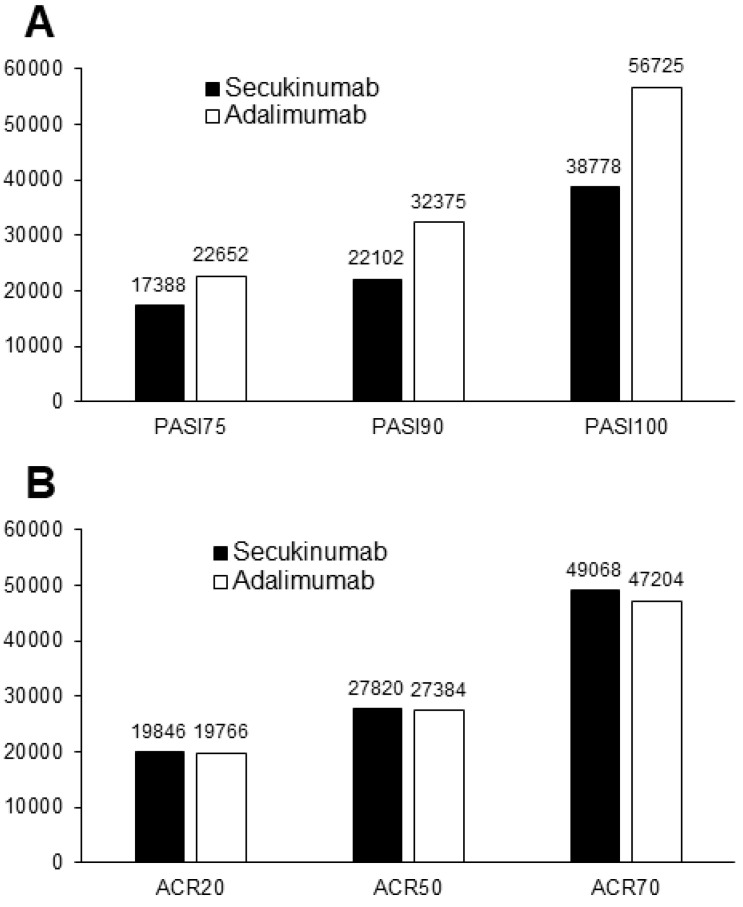
Cost per responder analysis for PASI 75/90/100 (**A**) and ACR 20/50/70 (**B**) in patients receiving secukinumab (black bars) and adalimumab (white bars) at week 52 (in Euro).

**Figure 2:**
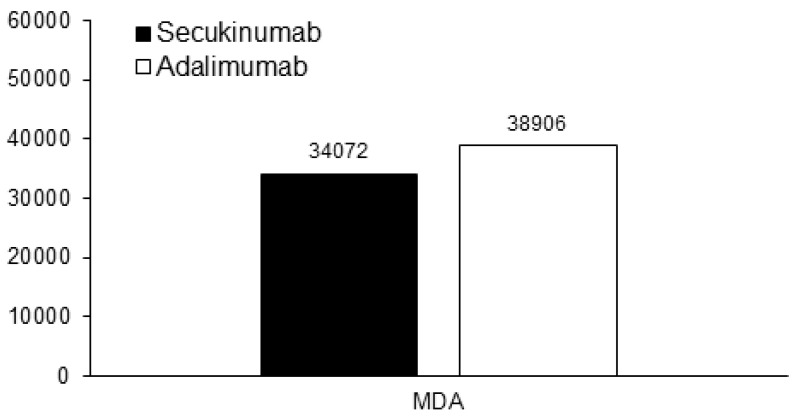
Cost per responder analysis for minimal disease activity in patients receiving secukinumab (black bars) and adalimumab (white bars) at week 52 (in Euro).

The cost per responder analysis for ACR 20/50/70 and PASI 75/90/100 response rate in patients receiving secukinumab and adalimumab at week 52 is reported in Figure 1. Considering the primary end point, the cost per responder for ACR 20 was €19,846 versus €19,766 for secukinumab and adalimumab, respectively. Considering the secondary end points, the costs per responder for ACR 50 and PASI 90 were €27,820 versus €27,384 and €22,102 versus €32,375 for secukinumab and adalimumab, respectively. Moreover, the cost per MDA responder was €34,072 for secukinumab and €38,906 for adalimumab (Figure 2). All the other end points of the study are reported in Supplementary Table S1. In particular, the cost per responder for ACR 70 was €49,068 for secukinumab versus €47,204 for adalimumab, respectively. The cost per responder for PASI 75 and PASI 100 was €17,388 versus €22,652 and €38,778 versus €56,725 for secukinumab and adalimumab, respectively. The scenario analyses considering different discount rates (from 5% to 35%) are reported in Supplementary Table S1–S8. Considering the secondary end points MDA and PASI 90, adalimumab would be more cost effective than secukinumab only in the case of a discount rate for adalimumab of 15% and 35% or greater, respectively.

## 4. Discussion

This study compared the economic value, using a cost per responder analysis, of secukinumab and adalimumab in patients with coexisting PsA and psoriasis after one year of treatment. The costs per responder associated with psoriasis outcomes were lower for secukinumab, whereas, regarding the PsA end points, the costs per responder were similar in terms of ACR 20 and ACR 50 response, and lower for adalimumab in terms of ACR 70 response. Considering the combined joint and skin outcome measure (MDA), the cost per responder was lower for secukinumab. 

Given the chronic nature of psoriatic disease and its long-term therapy, it is important to investigate the economic value of the different biologic agents, as even small differences in costs can be meaningful. With regard to PsA, biologic agents were shown to be cost effective compared to csDMARDs because, despite their higher costs, they are more effective in reducing the symptoms and signs of PsA, improving quality of life, and inhibiting structural radiological damage [18]. Among biologic agents approved for PsA, adalimumab and secukinumab represent two commonly prescribed agents with solid evidence supporting their efficacy [19]. Previous studies have compared their economic value in PsA. A British cost-effectiveness analysis that considered adult patients with active psoriatic arthritis who were naïve to TNF-α inhibitors, without concomitant moderate-to-severe psoriasis and who had responded inadequately to csDMARDs, found that secukinumab was associated with higher total costs but a greater number of quality-adjusted life years (QALYs) over the 40-year model time horizon compared to adalimumab, resulting in an ICER (incremental cost-effectiveness ratio) of £5680 per QALY gained for secukinumab versus adalimumab [20]. Furthermore, a Spanish cost-consequence analysis over a 2-year time frame found that the cost of initiating biologic therapy with secukinumab for PsA was 18–33% lower than that of adalimumab for ACR 20, 18–28% for ACR 50, and 16–23% for ACR 70 [21]. In a German cost-utility analysis over a lifetime horizon of secukinumab in patients with PsA with or without concomitant moderate to severe plaque psoriasis, secukinumab had a favorable ICER versus adalimumab in biologic-naïve patients without moderate to severe plaque psoriasis, while in those with concomitant moderate to severe plaque psoriasis and in those biologic-experienced, secukinumab was more effective and had a lower ICER than other bDMARDs, thus leading to extended dominance [22]. Conversely, an Argentinian cost-effectiveness analysis over a lifetime horizon of secukinumab versus other biologics for the treatment of PsA found that among biologic-naïve PsA patients without psoriasis, secukinumab dominated adalimumab, while among those biologic-naïve with psoriasis and those biologic-experienced, secukinumab was cost effective versus adalimumab [23]. Similarly, a Finnish cost-effectiveness analysis over a lifetime horizon of secukinumab versus other biologics in PsA found that secukinumab dominated adalimumab in biologic-naïve patients without moderate to severe psoriasis, while it was cost effective against adalimumab in biologic-naïve patients with moderate to severe psoriasis and biologic experienced patients [24]. Secukinumab also dominated adalimumab for the treatment of PsA in a Canadian cost-effectiveness analysis over a lifetime perspective that included both biologic-naïve and biologic-experienced patients [25]. Finally, an Irish matched adjusted indirect comparison analysis found that the cost per ACR 20 responder at week 48 is quite similar between secukinumab at the dose of 300 mg and adalimumab (i.e., EUR 29,092 and EUR 27,674, respectively), but lower for secukinumab at a dose of 150 mg (i.e., EUR 13,147 vs. 27,674, respectively) [26]. 

The present study failed to detect the higher cost effectiveness of secukinumab over adalimumab in PsA that was found in previous studies. However, this may be explained by methodological differences: this study used a cost per responder model based on efficacy data from a phase 3 trial with a 1-year time horizon, whereas previous studies employed cost effectiveness analyses over much longer time horizons. Indeed, short-term cost calculations tend to penalize drugs with a more expensive induction phase [27], such as in the case of secukinumab.

Conversely, regarding plaque psoriasis, the findings of the present study are in line with previous studies. In particular, a German 52-week cost per responder model that compared secukinumab with other biologics for the treatment of plaque psoriasis found that secukinumab had the lowest cost per sustained PASI 90 responder (€22,690) compared with adalimumab, etanercept, infliximab, and ustekinumab [28]. Of note, other anti-IL-17 inhibitors and IL-23 inhibitors were not included in the analysis, as they were not available yet. Similarly, a Spanish 2-year cost-consequence study found that secukinumab had a lower cost per responder for the treatment of moderate-to-severe psoriasis than adalimumab, ustekinumab, infliximab, and etanercept, and that treatment sequences starting with secukinumab were the most cost efficient [27]. Furthermore, a 2-year German payer perspective analysis that assessed the impact of placing secukinumab in psoriasis treatment sequencing with adalimumab, etanercept, infliximab, and ustekinumab found that using secukinumab as first-line biologic treatment was cost effective compared with initiating other biologic agents [29]. Conflicting findings were reported in a recent Italian cost per responder model based on the CANOVA (EffeCtiveness of biologic treAtmeNts for plaque psOriasis in Italy: an obserVAtional longitudinal study of real-life clinical practice) real-world study [30,31]. In that study, the costs per PASI 75/90/100 responder at 52 weeks were higher for secukinumab than adalimumab originator: €19,932 vs. €18,491, €23,978 vs. €22,755 and €33,419 vs. €31,378 respectively [31]. However, the number of patients on secukinumab (*n* = 274) and adalimumab (*n* = 87) were unbalanced [31]. Further pharmacoeconomic studies based on larger real-world samples are needed to draw definite conclusions.

Of note, secukinumab also presents further advantages over adalimumab other than its cost effectiveness. First, its higher efficacy allows a higher percentage of patients to reach PASI 90 or PASI 100 [32,33]. This has important long-term economic implications given that higher PASI responses were shown to be associated with reduced total work productivity impairment in patients with moderate-to-severe psoriasis [34]. Indeed, secukinumab was found to significantly reduce work impairment and psoriasis-associated indirect costs compared with ustekinumab and etanercept [35]. Furthermore, secukinumab has a more favorable safety profile and fewer contraindications than adalimumab [36,37]; for example, secukinumab carries lower risk of serious infections, demyelinating diseases, and reactivation of latent tuberculosis or hepatitis B, making it a preferred choice in some patients. 

There are limitations to this study that should be noted. First, ex-factory wholesale purchase prices with the mandatory discounts (−5%; −5%) were used in the cost responder model, yet retail discounts may vary widely (from 20% to 80%) and could change the economic evaluation. Furthermore, adalimumab originator was considered for the model because it was the one tested in the EXCEED study. However, adalimumab biosimilars are available and have shown a comparable efficacy to the originator, while their cost is significantly lower than that of the originator [38,39,40]. Another limitation of the study is that we included in this analysis only drug acquisition costs without considering the costs of the visits and laboratory screening/monitoring. However, these costs can be reasonably supposed to be quite similar between the two treatments according to the EuroGuiDerm guideline on psoriasis [3]. Finally, we did not consider that in the EXCEED study the dropout rates were 5.5% for secukinumab and 17.8% for adalimumab, respectively.

## 5. Conclusions

The costs per responder associated with the ACR 20 and ACR 50 end points were similar for adalimumab compared to secukinumab; conversely, for the psoriasis and composite end points, they were lower for secukinumab.

## Figures and Tables

**Table 1:** Efficacy data of secukinumab and adalimumab at week 52 (%) from the EXCEED study.

	Secukinumab	Adalimumab	P ^
ACR 20	76.4	68.3	0.07
ACR 50	54.5	49.3	0.22
ACR 70	30.9	28.6	0.29
Minimal disease activity	44.5	34.7	0.14
PASI 75	87.2	59.6	0.01
PASI 90	68.6	41.7	0.01
PASI 100	39.1	23.8	0.01

ACR: American college of rheumatology (% improvement); PASI: psoriasis area and severity index % improvement). ^ the EXCEED study was designed for investigating the superiority of secukinumab versus adalimumab. The 52-week primary endpoint was the ACR 20, and the secondary endpoints were ACR 50 and PASI 90.

**Table 2:** Number of administrations of secukinumab and adalimumab at week 52.

Drug	Dosage	Number of Administrations at Week 52
Secukinumab	300 mg by subcutaneous injection at weeks 0, 1, 2, 3, and 4 followed by 300 mg every 4 weeks	16
Adalimumab	80 mg by subcutaneous injection at week 0, followed by 40 mg every other week beginning one week after initial dose	27

**Table 3:** Costs of secukinumab and adalimumab (in Euro).

Drug (Trade Name)	Ex-Factory Price per Package ^	Discount	Discounted Price per Package	Costs at 16 Weeks	Costs at 52 Weeks
Secukinumab 150 mg	1050.0	5% and 5%	947.6	6633.4	15162.1
Adalimumab 40 mg	1068.5	5% and 5%	964.3	4821.6	13500.5

^ https://www.gazzettaufficiale.it/eli/id/2016/11/11/16A07913/sg; https://www.gazzettaufficiale.it/eli/id/2021/07/28/21A04520/SG#:~:text=Prezzo%20al%20pubblico%20(iva%20inclusa,E%20(in%20base%2010) (accessed on 2 March 2022).

## Data Availability

All data generated during this study are included in this published article (and its Supplementary Information Files).

## Supplementary Materials

The following supporting information can be downloaded at https://www.mdpi.com/article/10.3390/vaccines10050646/s1: Table S1: Cost per responder of secukinumab and adalimumab (in Euro) at week 52; Table S2: Scenario analysis of the cost per ACR 20 responder at 52 weeks; Table S3: Scenario analysis of the cost per ACR 50 responder at 52 weeks; Table S4: Scenario analysis of the cost per ACR 70 responder at 52 weeks; Table S5: Scenario analysis of the cost per MDA responder at 52 weeks; Table S6: Scenario analysis of the cost per PASI 75 responder at 52 weeks; Table S7: Scenario analysis of the cost per PASI 90 responder at 52 weeks; Table S8: Scenario analysis of the cost per PASI 100 responder at 52 weeks.

## References

[1] Damiani G., Bragazzi N.L., Karimkhani Aksut C., Wu D., Alicandro G., McGonagle D., Guo C., Dellavalle R., Grada A., Wong P. (2021). The global, regional, and national burden of psoriasis: Results and insights from the global burden of disease 2019 study. Front. Med..

[2] Colombo G., Altomare G., Peris K., Martini P., Quarta G., Congedo M., Costanzo A., Di Cesare A., Lapucci E., Chimenti S. (2008). Moderate and severe plaque psoriasis: Cost-of-illness study in Italy. Ther. Clin. Risk Manag..

[3] Nast A., Gisondi P., Ormerod A.D., Saiag P., Smith C., Spuls P.I., Arenberger P., Bachelez H., Barker J., Dauden E. (2015). European S3-Guidelines on the systemic treatment of psoriasis vulgaris--Update 2015--Short version--EDF in cooperation with EADV and IPC. J. Eur. Acad. Dermatol. Venereol..

[4] Esposti L.D., Perrone V., Sangiorgi D., Buda S., Andretta M., Rossini M., Girolomoni G. (2018). Analysis of drug utilization and health care resource consumption in patients with psoriasis and psoriatic arthritis before and after treatment with biological therapies. Biologics.

[5] Gottlieb A., Merola J.F. (2020). Psoriatic arthritis for dermatologists. J. Dermatolog. Treat..

[6] Amin M., Lee E.B., Tsai T.F., Wu J.J. (2020). Psoriasis and co-morbidity. Acta Derm Venereol..

[7] Kulkarni A.S., Balkrishnan R., Camacho F.T., Anderson R.T., Feldman S.R. (2004). Medication and health care service utilization related to depressive symptoms in older adults with psoriasis. J. Drugs Dermatol..

[8] Oliveira Mde F., Rocha Bde O., Duarte G.V. (2015). Psoriasis: Classical and emerging comorbidities. An. Bras. Dermatol..

[9] Wu J.J., Feldman S.R., Ko J., Marangell L.B. (2018). Epidemiology of mental health comorbidity in psoriasis. J. Dermatolog. Treat..

[10] Singh S., Taylor C., Kornmehl H., Armstrong A.W. (2017). Psoriasis and suicidality: A systematic review and meta-analysis. J. Am. Acad. Dermatol..

[11] Armstrong A.W., Schupp C., Wu J., Bebo B. (2012). Quality of life and work productivity impairment among psoriasis patients: Findings from the National Psoriasis Foundation survey data 2003–2011. PLoS ONE.

[12] Gisondi P., Geat D., Pizzolato M., Girolomoni G. (2019). State of the art and pharmacological pipeline of biologics for chronic plaque psoriasis. Curr. Opin. Pharmacol..

[13] McInnes I.B., Behrens F., Mease P.J., Kavanaugh A., Ritchlin C., Nash P., Masmitja J.G., Goupille P., Korotaeva T., Gottlieb A.B. (2020). Secukinumab versus adalimumab for treatment of active psoriatic arthritis (EXCEED): A double-blind, parallel-group, randomised, active-controlled, phase 3b trial. Lancet.

[14] Gottlieb A.B., Merola J.F., Reich K., Behrens F., Nash P., Griffiths C.E.M., Bao W., Pellet P., Pricop L., McInnes I.B. (2021). Efficacy of secukinumab and adalimumab in patients with psoriatic arthritis and concomitant moderate-to-severe plaque psoriasis: Results from EXCEED, a randomized, double-blind head-to-head monotherapy study. Br. J. Dermatol..

[15] Gossec G., McGonagle D., Korotaeva T., Lubrano E., de Miguel E., Østergaard M., Behrens F. (2018). Minimal Disease Activity as a Treatment Target in Psoriatic Arthritis: A Review of the Literature. J. Rheum..

[16] https://www.gazzettaufficiale.it/eli/id/2016/11/11/16A07913/sg..

[17] https://www.gazzettaufficiale.it/eli/id/2021/07/28/21A04520/SG#:~:text=Prezzo%20al%20pubblico%20.

[18] D’Angiolella L.S., Cortesi P.A., Lafranconi A., Micale M., Mangano S., Cesana G., Mantovani L.G. (2018). Cost and cost effectiveness of treatments for psoriatic arthritis: A systematic literature review. Pharmacoeconomics.

[19] Ogdie A., Coates L.C., Gladman D.D. (2020). Treatment guidelines in psoriatic arthritis. Rheumatology.

[20] Buchanan V., Sullivan W., Graham C., Miles L., Jugl S.M., Gunda P., Halliday A., Kirkham B. (2018). Cost effectiveness of secukinumab for the treatment of active psoriatic arthritis in the UK. Pharmacoeconomics.

[21] Jiménez-Morales A., Cáliz R., Aceituno S., Prades M., Blanch C. (2021). A cost-consequence analysis of the preferential use of secukinumab versus adalimumab for the treatment of psoriatic arthritis. Reumatol. Clin..

[22] Gandjour A., Ostwald D.A. (2020). Cost effectiveness of secukinumab versus other biologics and apremilast in the treatment of active psoriatic arthritis in Germany. Appl. Health Econ. Health Policy.

[23] Aiello E., Bianculli P.M., Bhattacharyya D., Gunda P., Citera G. (2019). Cost-effectiveness of secukinumab versus other biologics in the treatment of psoriatic arthritis: An Argentinean perspective. Value Health Reg. Issues.

[24] Purmonen T., Puolakka K., Bhattacharyya D., Jain M., Martikainen J. (2018). Cost-effectiveness analysis of secukinumab versus other biologics and apremilast in the treatment of active psoriatic arthritis: A Finnish perspective. Cost Eff. Resour. Alloc..

[25] Goeree R., Chiva-Razavi S., Graham C.N., Miles L., Nikoglou E., Jugl S.M., Gladman D.D. (2018). Cost-effectiveness analysis of secukinumab for the treatment of active psoriatic arthritis: A Canadian perspective. J. Med. Econ..

[26] Gunda P., Nikoglou E., Jugl S.M., Murphy A. (2017). A Cost Per Responder Analysis of Secukinumab Vs. Adalimumab Based on a Matching-Adjusted Indirect Comparison of Efficacy Data for the Treatmetn of Psoriatic Arthritis at 48 Weeks from the Irish Payer Perspective. Value Health.

[27] Puig L., Notario J., Jimenez-Morales A., Moreno-Ramírez D., López-Ferrer A., Gozalbo I., Prades M., Lizán L., Blanch C. (2017). Secukinumab is the most efficient treatment for achieving clear skin in psoriatic patients: A cost-consequence study from the Spanish National Health Service. J. Dermatol. Treat..

[28] Augustin M., McBride D., Gilloteau I., O’Neill C., Neidhardt K., Graham C.N. (2018). Cost-effectiveness of secukinumab as first biologic treatment, compared with other biologics, for moderate to severe psoriasis in Germany. J. Eur. Acad. Dermatol. Venereol..

[29] Augustin M., Krieger T., McBride D., Graham C.N., Melzer N., Kneidl J., Neidhardt K. (2016). Cost-effectiveness of secukinumab as first biologic treatment for psoriasis compared with initiating other biologic therapy in Germany. Value Health.

[30] Colombo D., Bianchi L., Fabbrocini G., Corrao S., Offidani A., Stingeni L., Costanzo A., Pellacani G., Peris K., Bardazzi F. (2022). Real-world evidence of biologic treatments in moderate-severe psoriasis in Italy: Results of the CANOVA (EffeCtiveness of biologic treAtmeNts for plaque psOriasis in Italy: An obserVAtional longitudinal study of real-life clinical practice) study. Dermatol. Ther..

[31] Zagni E., Bianchi L., Fabbrocini G., Corrao S., Offidani A., Stingeni L., Costanzo A., Pellacani G., Peris K., Bardazzi F. (2021). A real-world economic analysis of biologic therapies for moderate-to-severe plaque psoriasis in Italy: Results of the CANOVA observational longitudinal study. BMC Health Serv. Res..

[32] Sbidian E., Chaimani A., Garcia-Doval I., Doney L., Dressler C., Hua C., Hughes C., Naldi L., Afach S., Le Cleach L. (2021). Systemic pharmacological treatments for chronic plaque psoriasis: A network meta-analysis. Cochrane Database Syst. Rev..

[33] Reich K., Warren R.B., Coates L.C., Di Comite G. (2020). Long-term efficacy and safety of secukinumab in the treatment of the multiple manifestations of psoriatic disease. J. Eur. Acad. Dermatol. Venereol..

[34] Lebwohl M., Soliman A.M., Yang H., Wang J., Freimark J., Puig L. (2022). Impact of PASI response on work productivity and the effect of risankizumab on indirect costs using machine learning in patients with moderate-to-severe psoriasis. J. Dermatolog. Treat..

[35] Warren R.B., Halliday A., Graham C.N., Gilloteau I., Miles L., McBride D. (2018). Secukinumab significantly reduces psoriasis-related work impairment and indirect costs compared with ustekinumab and etanercept in the United Kingdom. J. Eur. Acad. Dermatol. Venereol..

[36] Blauvelt A. (2016). Safety of secukinumab in the treatment of psoriasis. Expert Opin. Drug Saf..

[37] Thatiparthi A., Martin A., Liu J., Egeberg A., Wu J.J. (2021). Biologic treatment algorithms for moderate-to-severe psoriasis with comorbid conditions and special populations: A review. Am. J. Clin. Dermatol..

[38] Puig L., López-Ferrer A. (2019). Biosimilars for the treatment of psoriasis. Expert Opin. Biol. Ther..

[39] Gisondi P., Geat D., Conti A., Dapavo P., Piaserico S., De Simone C., Bianchi L., Costanzo A., Malagoli P., Malara G. (2020). TNF-α inhibitors biosimilars as first line systemic treatment for moderate-to-severe chronic plaque psoriasis. Expert Rev. Clin. Immunol..

[40] Barker J., Girolomoni G., Egeberg A., Goncalves J., Pieper B., Kang T. (2020). Anti-TNF biosimilars in psoriasis: From scientific evidence to real-world experience. J. Dermatolog. Treat..

