# Supplementary material for: Cost per Responder Analysis of Secukinumab versus Adalimumab in the Treatment of Psoriatic Disease
Source: Vaccines (Basel). 2022 Apr 20;10(5):646. doi: 10.3390/vaccines10050646 (PMC9142895; doi:10.3390/vaccines10050646)
Supplement: Supplementary file 1 [file vaccines-10-00646-s001.zip › vaccines-1673112-supplementary.pdf]

Supplementary Table S1. Cost per responder of secukinumab and adalimumab (in Euro) at week 52

	Secukinumab (Cosentyx®)	Adalimumab (Humira®)
ACR 20	19846	19766
ACR 50	27820	27384
ACR 70	49068	47204
Minimal disease activity	34072	38906
PASI 75	17388	22652
PASI 90	22102	32375
PASI 100	38778	56725

Supplementary Table S2. Scenario analysis of the cost per ACR20 responder at 52 weeks.

	Secukinumab discount rate	5%	10%	15%	20%	25%	30%	35%
Adalimumab discount rate	Cost per responder (Euro)	18853.37	17861.09	16868.81	15876.52	14884.24	13891.96	12899.68
5%	18778.12							
10%	17789.80							
15%	16801.48							
20%	15813.15							
25%	14824.83							
30%	13836.51							
35%	12848.19							

Green: cost per responder of secukinumab < cost per responder of adalimumab. Red: cost per responder of secukinumab > cost per responder of adalimumab.

Supplementary Table S3. Scenario analysis of the cost per ACR50 responder at 52 weeks.

	Secukinumab discount rate	5%	10%	15%	20%	25%	30%	35%
Adalimumab discount rate	Cost per responder (Euro)	26429.31	25038.30	23647.28	22256.26	20865.25	19474.23	18083.21
5%	26015.12							
10%	24645.91							
15%	23276.69							
20%	21907.47							
25%	20538.26							
30%	19169.04							
35%	17799.82							

Green: cost per responder of secukinumab < cost per responder of adalimumab. Red: cost per responder of secukinumab > cost per responder of adalimumab.

Supplementary Table S4. Scenario analysis of the cost per ACR70 responder at 52 weeks.

	Secukinumab discount rate	5%	10%	15%	20%	25%	30%	35%
Adalimumab discount rate	Cost per responder (Euro)	46614.81	44161.40	41707.99	39254.58	36801.17	34347.75	31894.34
5%	44844.25							
10%	42484.03							
15%	40123.80							
20%	37763.58							
25%	35403.36							
30%	33043.13							
35%	30682.91							

Green: cost per responder of secukinumab < cost per responder of adalimumab. Red: cost per responder of secukinumab > cost per responder of adalimumab.

Supplementary Table S5. Scenario analysis of the cost per MDA responder at 52 weeks.

	Secukinumab discount rate	5%	10%	15%	20%	25%	30%	35%
Adalimumab discount rate	Cost per responder (Euro)	32368.49	30664.88	28961.28	27257.67	25554.07	23850.46	22146.86
5%	36960.97							
10%	35015.65							
15%	33070.34							
20%	31125.03							
25%	29179.71							
30%	27234.40							
35%	25289.08							

Green: cost per responder of secukinumab < cost per responder of adalimumab. Red: cost per responder of secukinumab > cost per responder of adalimumab.

Supplementary Table S6. Scenario analysis of the cost per PASI75 responder at 52 weeks.

	Secukinumab discount rate	5%	10%	15%	20%	25%	30%	35%
Adalimumab discount rate	Cost per responder (Euro)	16518.32	15648.94	14779.55	13910.17	13040.78	12171.39	11302.01
5%	21519.22							
10%	20386.63							
15%	19254.04							
20%	18121.45							
25%	16988.86							
30%	15856.27							
35%	14723.68							

Green: cost per responder of secukinumab < cost per responder of adalimumab. Red: cost per responder of secukinumab > cost per responder of adalimumab.

Supplementary Table S7. Scenario analysis of the cost per PASI90 responder at 52 weeks.

	Secukinumab discount rate	5%	10%	15%	20%	25%	30%	35%
Adalimumab discount rate	Cost per responder (Euro)	20997.05	19891.94	18786.83	17681.73	16576.62	15471.51	14366.40
5%	30756.49							
10%	29137.73							
15%	27518.96							
20%	25900.20							
25%	24281.44							
30%	22662.68							
35%	21043.91							

Green: cost per responder of secukinumab < cost per responder of adalimumab. Red: cost per responder of secukinumab > cost per responder of adalimumab.

Supplementary Table S8. Scenario analysis of the cost per PASI100 responder at 52 weeks.

	Secukinumab discount rate	5%	10%	15%	20%	25%	30%	35%
Adalimumab discount rate	Cost per responder (Euro)	36838.81	34899.93	32961.04	31022.16	29083.27	27144.39	25205.50
5%	53888.47							
10%	51052.24							
15%	48216.00							
20%	45379.76							
25%	42543.53							
30%	39707.29							
35%	36871.06							

Green: cost per responder of secukinumab < cost per responder of adalimumab. Red: cost per responder of secukinumab > cost per responder of adalimumab.
